# A comparative evaluation of corticotomy-assisted and conventional maxillary anterior en-masse retraction: A clinical study

**DOI:** 10.6026/973206300223400

**Published:** 2026-06-30

**Authors:** Ruchi Raval, Surendra Lodha, Anand Mohan, Sanket Sheth, Sangita Bhalodi, Tejomaya Shastry

**Affiliations:** 1 Department of Periodontology, K. M. Shah Dental College & Hospital, Sumandeep Vidyapeeth Deemed to be University, Vadodara, Gujarat, India; 2 Private Dental Practitioner, Umrao Dental Care and Orthodontic Center, Bhilwara, Rajasthan, India; 3 Department of Dentistry, Nalanda Medical College & Hospital, Agamkuan, Patna, Bihar, India; 4 Private Dental Practitioner, Bombay Dental Specialities, Mumbai, Maharashtra, India; 5 Private Dental Practitioner, Restore Dental Treatment Centre, Rajkot, Gujarat, India; 6 Specialist Prosthodontist, Dr Joy Dental Clinic L.L.C., Dubai, United Arab Emirates

**Keywords:** Corticotomy, orthodontic tooth movement (OTM), regional accelerated phenomenon, periodontally accelerated osteogenic orthodontics (PAOO), orthodontic space closure

## Abstract

Orthodontic patients often experience prolonged treatment duration and anchorage loss during maxillary anterior en-masse retraction. This drawback has prompted the need for techniques that can accelerate tooth movement. Therefore, it is of interest to evaluate the effectiveness of corticotomy-assisted versus conventional maxillary anterior en-masse retraction in adult patients. It was conducted in the Department of Orthodontics and Dentofacial Orthopaedics, Manipal College of Dental Sciences, Manipal, Karnataka, India. Twelve patients with Class I bimaxillary protrusion were divided equally into two groups and treated with identical orthodontic mechanics. The rate of tooth movement, anchorage loss and incisor torque control were assessed at defined time intervals during space closure. Corticotomy-assisted retraction showed a significantly higher rate of tooth movement and reduced overall treatment time with less anchorage loss. No significant difference in torque control was observed between the two treatment modalities.

## Background:

The goal of orthodontic treatment is to improve the patient's quality of life through the enhancement of dento-facial function and aesthetics. The duration of orthodontic treatment is an important issue, particularly for adults. Lengthy orthodontic treatment time has been linked to an increased risk of root resorption, gingival inflammation, decalcification and dental caries [1, 2]. Therefore, reducing the treatment time is an appropriate goal, which requires increasing the rate of tooth movement. Several studies have demonstrated that successful acceleration of tooth movement can be produced by local injection of prostaglandins, vitamin D3 and osteocalcin, application of pulsed electromagnetic fields and direct electric current to selected teeth [3]. However, these methods are not without undesirable side effects. Rapid orthodontic tooth movement can be attained through a combination of orthodontic treatment and surgical alveolar corticotomies [1, 2]. Corticotomy is defined as any intentional surgical injury to cortical bone. In adult patients, this technique decreases the resistance of the dense cortical bone, thereby reducing the treatment time significantly [4]. Therefore, it is of interest to report and compare corticotomy-assisted and conventional maxillary anterior en-masse retraction by evaluating the rate of tooth movement, anchorage loss and torque control.

## Materials and Methods:

The study was conducted in the Department of Orthodontics and Dentofacial Orthopaedics, Manipal College of Dental Sciences, Manipal, Karnataka, India. Research approval was obtained from the Institutional Ethical Committee (IEC). The study included patients aged 22 ± 3 years. All patients had less than 4 mm of crowding and a normo-divergent skeletal pattern. Anterior proclination was present, with an upper incisor-NA angle between 24° and 28°. Gender differences were not considered. Patients were excluded if they had systemic conditions such as blood dyscrasias. Those with malformed or missing teeth were excluded. Any history of previous orthodontic treatment was an exclusion factor. High anchorage cases were not included. Patients with poor oral hygiene, probing depths greater than 3 mm, loss of periodontal attachment, or radiographic evidence of bone loss were also excluded. Twelve patients with Class I bimaxillary protrusion requiring the extraction of all first premolars were selected. Participants were divided into two groups: conventional en-masse maxillary anterior retraction (n=6) and corticotomy-assisted retraction (n=6) based on consent, following strict inclusion and exclusion criteria. All subjects received standardised fixed appliance therapy and identical retraction mechanics. In Group 2, corticotomy was performed after levelling and alignment under local anaesthesia. Sulcular incisions were made on the buccal and palatal aspects of the maxillary anterior segment, extending from the mesial line angle of the second premolar on one side to the contralateral side. Full-thickness trapezoidal buccal and envelope palatal flaps were raised to expose the alveolar bone. Vertical inter-radicular corticotomy cuts were placed 2-3 mm apical to the alveolar crest and extended 2-3 mm beyond the estimated root apices. Cuts were done using a No. 701 surgical bur under copious saline irrigation, connected by scalloped horizontal cuts. Multiple round perforations were created within the corticotomized areas and filled with synthetic hydroxyapatite graft (0.25-0.5 mL per tooth). Care was taken to limit cuts to the cortical bone with minimal trabecular penetration. Primary flap closure was achieved with non-resorbable interrupted sutures, followed by periodontal dressing and routine post-operative medication. The parameters measured included the rate of tooth movement, anchorage control and torque control. The rate of tooth movement was assessed by recording the total time required for maxillary anterior en-masse retraction and by measuring space closure at 2, 4, 8 and 12 weeks. Changes were evaluated using a digital vernier calliper in the inter-bracket distance between the canine and second premolar. Anchorage control was evaluated by measuring the mesial movement of the maxillary first molars on lateral cephalograms. The pterygoid vertical plane was used as the reference line. Right and left molars were differentiated using L-shaped stainless-steel markers. Torque control was assessed on lateral cephalograms by measuring the angular relationship between the long axis of the maxillary incisor and the nasal floor. This assessment was performed according to the COGS analysis.

## Results: 

Total time required and total amount of retraction for each patient are shown in Table 1. Average time required for maxillary en-mass retraction was 25.83 ± 1.72 weeks in group 1, whereas 12.5 ± 0.55 weeks in group 2 (Table 1). Mean rate of tooth movement was 0.56 ± 0.02 mm/week and 1.16 ± 0.05 mm/week in group 1 and group 2, respectively (Table 2). An independent sample t-test showed that the rate of tooth movement was significantly higher in group 2 than in group 1 (p < 0.001). The rate of tooth movement at weekly intervals in group 2 varied significantly from 2 weeks to 12 weeks. Post hoc test showed that the tooth movement was significantly higher at 6-to-8-week intervals (p<0.001) (Table 3). The mean anchorage loss was 2.33 ± 0.52 mm and 1.25 ± 0.42 mm in groups 1 and 2, respectively (Table 4). An independent sample t-test revealed that the mean anchorage loss was significantly higher for group 1 than for group 2 (p<0.003). Torque control for both groups is shown in Table 5. Independent sample t-test showed no significant difference in the mean torque loss between group 1 and 2 (p = 0.707).

## Discussion:

Interdisciplinary orthodontic tooth movement (OTM) integrates tissue engineering with periodontal regenerative surgery. This approach aims to accelerate tooth movement while minimising adverse effects. These include root resorption, relapse, inadequate basal bone and bacterial time-load factors such as infection [5]. Periodontally accelerated osteogenic orthodontics (PAOO) combines selective decortication-facilitated orthodontics with alveolar augmentation. This technique allows teeth to be moved 2-3 times farther in one-third to one-fourth of the time required for conventional orthodontic treatment [4, 6]. The study hypothesised that corticotomy could reduce treatment duration and resistance from dense cortical bone, thereby accelerating tooth movement [4]. Standard corticotomy was performed in both buccal and palatal cortical plates with sub-apical cuts only [7]. This was based on the evidence by Sebaoun *et al.* [8] that selective alveolar decortication produces a strong stimulatory effect on both catabolic (resorptive) and anabolic (formative) periodontal processes. Bone modelling peaked at three weeks post-surgery, with a threefold increase in osteoclastic activity and bone apposition. Tissue turnover normalised by 11 weeks and remained localised to the injured area. These findings also support the hypothesis of Wilcko *et al.* [9]. En masse retraction was selected because two-step space closure may prolong treatment duration [10], and individual canine retraction often results in tipping and rotation requiring additional correction [11]. En masse retraction was performed using frictionless mechanics, which provides more controlled tooth movement with reduced tipping and extrusion compared to friction mechanics [12]. Anchorage control is critical in extraction cases to prevent unwanted molar mesialization. While temporary anchorage devices (TADs) offer absolute anchorage, their use is limited by anatomical constraints. PAOO causes a transient reduction in cortical resistance, increasing differential tooth movement. Non-corticotomized teeth thus provide improved relative anchorage. A T-loop was selected for retraction due to its favourable moment-to-force (M/F) ratio of 7:1 at 4 mm activation and 9:1 at 2 mm. Continuous T-loops are safer and preserve the arch form better than segmental T-loops [13]. A β moment of 40° was incorporated without an α moment to enhance anchorage through the mesial root moment of the posterior teeth [14]. This generated an intrusive force on anterior teeth and an extrusive force on molars, which was counteracted using a transpalatal arch (TPA) in both groups. The study results showed that PAOO significantly accelerated tooth movement. Group 2 demonstrated a statistically significant increase in tooth movement rate from 2 to 12 weeks (p<0.001), with peak movement between 6 and 8 weeks. These findings are consistent with animal studies by Lino *et al.* [15] and Mostafa *et al.* [16] and corroborate clinical observations by Wilcko *et al.* [9] who reported significant reductions in treatment time with corticotomy-assisted orthodontics. Few studies [10, 10] have evaluated anchorage loss during anterior en-masse retraction in humans, and none have assessed anchorage loss with corticotomy-assisted maxillary retraction. This study found significantly greater anchorage loss in group 1 compared to group 2 (p<0.003). Reduced molar mesial movement in the corticotomy group may be attributed to increased differential movement, where activated teeth move readily and non-activated teeth provide better relative anchorage. Research advancements on corticotomy have shifted from classic, flap-based surgery to minimally invasive, digitally guided procedures that aim to keep the acceleration benefits while reducing morbidity. In a case report presented in 2025 by Tsukiboshi *et al.*, a 32-year-old female patient, a digitally guided Suya corticotomy was performed using a CBCT- and IOS-based 3D-printed surgical guide. Piezoelectric osteotomies and guided chisel application followed the pre-planned vertical and apical cuts with high accuracy and controlled fracturization. Post-operative CBCT confirmed safe osteotomy positioning between roots with no root contact or damage, demonstrating reliable clinical reproducibility [18]. A study using 3D finite element analysis and CBCT was done on a 24-year-old male by Hou *et al.* in 2025. Detailed models of the maxilla, teeth, PDLs, aligners and attachments were created for simulating anterior retraction with clear aligners. Four models with different corticotomy designs included no corticotomy, bilateral horizontal + vertical, unilateral horizontal + vertical and unilateral vertical. Bilateral horizontal and vertical corticotomy produced the greatest tooth displacement and stress redistribution, followed by unilateral horizontal and vertical incisions. Unilateral vertical corticotomy showed minimal differences compared with no corticotomy [19]. A meta-analysis in 2024 by Zhou *et al.* systematically reviewed clinical trials from PubMed, Cochrane and Embase (as of May 2023) that evaluated surgically facilitated orthodontic strategies. Nineteen studies (each with≥10 subjects) were included, and outcomes were analysed using WMD/SMD with confidence intervals. Results showed corticotomy significantly reduced alignment time and accelerated canine movement compared with conventional orthodontics. PAOO further shortened total treatment duration and significantly increased alveolar bone thickness, supporting its clinical effectiveness [20]. A review by Guarnieri *et al.* in 2023 highlighted the application of computer-guided surgery - CAD/CAM in orthodontics, particularly for miniscrews and corticotomy techniques. A PubMed search identified 27 relevant articles, demonstrating that digital planning improved precision, predictability and efficiency, even for less experienced clinicians. Overall, digital workflows facilitated faster and safer procedures and allowed potential issues to be identified and corrected preoperatively [21]. The regional acceleratory phenomenon (RAP), involves both hard and soft tissues. With this aim, a split-mouth study was done by Gopalakrishnan *et al.* in 2023 to compare a soft tissue flap procedure with conventional corticotomy in 40 participants. Although corticotomy showed greater canine angulation and differences in rotational changes, these findings were not statistically significant. Overall, both approaches demonstrated comparable effects on tooth movement and dentoalveolar changes [22]. A randomised clinical trial by Khlef and Hajeer in 2022 aimed to compare traditional corticotomy (TC) and flapless corticotomy (FC) in accelerating maxillary anterior en-masse retraction in adults with Class II division 1 malocclusion. The TC group showed a significantly higher rate and greater amount of en masse retraction during the first three months compared with the FC group. Dental arch changes were comparable between groups, while periodontal indices were significantly worse in the TC group, with no gingival recession or loss of pulp vitality in either group. Overall, both techniques were clinically effective, with flapless corticotomy emerging as a less invasive yet reliable alternative to traditional corticotomy [23]. A split-mouth randomised clinical trial aimed to compare the efficacy of micro-osteoperforations (MOP) and corticotomy in accelerating maxillary canine retraction. It was done by Perez-Cisneros *et al.* in 2023 over 3 months; both techniques produced similar amounts of canine retraction at the incisal and medial levels, with no statistically significant differences between groups. The results indicate that MOPs and corticotomy are comparably effective in enhancing maxillary canine retraction [24]. In a PRISMA-based systematic review of 13 randomised clinical trials by Faccioni *et al.* in 2025 [25], four studies reported statistically significant differences between surgical techniques. Abbas *et al.* in 2016 [26] and Fernandes *et al.* in 2021 [27] found a higher rate of canine movement with corticotomy compared to piezocision. Chandra *et al.* [28] reported shorter treatment duration with PAOO versus corticotomy in 2019, and Khlef *et al.* (2022) [23] observed a higher rate of en masse retraction with traditional corticotomy compared to flapless corticotomy. The remaining studies found no significant differences, indicating that no single technique demonstrated consistent superiority. Corticotomy has shown promising results in many cases similar to ours. Limitations of our research include the need for frequent activations, increased cost and post-operative discomfort.

## Conclusion:

Corticotomy-assisted orthodontics is a promising adjunctive technique, particularly for adult patients. The study confirms faster tooth movement and reduced anchorage loss compared to conventional methods. However, long-term studies with larger sample sizes are required to further evaluate its benefits and limitations.

## Figures and Tables

**Table 1:** Total time required and amount of retraction

**Patient**	**Group 1**		**Group 2**	
	Total space closed (mm.)	Total time (weeks)	Total space closed(mm.)	Total time (weeks)
1	14.3	25	14.2	12
2	13.7	23	14.7	12
3	15	28	14.3	12
4	14.6	27	14.4	13
5	14.3	26	14.5	13
6	14.8	26	14.7	13

**Table 2:** Mean rate of tooth movement

	**Group**				**p-value**
	1		2		
	Mean	SD	Mean	SD	
Rate of tooth movement	0.56	0.02	1.16	0.05	<0.001 Sig.

**Table 3:** Mean rate of tooth movement at weekly intervals in group 2

**Intervals**	**Mean**	**SD**	**N**	**p-value**	**Post hoc test**
TM 0-2	1.15	0.12	6	<0.001	3>1,2
TM 2-4	1.43	0.1	6	Sig.	4>1,2,3
TM 4-6	3.4	0.17	6		5>1,2,3
TM 6-8	4.1	0.17	6		
TM 8-12	3.97	0.23	6		

**Table 4:** Mean anchorage loss

	**Group**				**p-value**
	1		2		
	Mean	SD	Mean	SD	
anchorage loss	2.33	0.52	1.25	0.42	0.003 Sig.

**Table 5:** Mean torque deviation

	**Group**				**p-value**
	1		2		
	Mean	SD	Mean	SD	
Torque control	-7.58	7.74	-5.83	7.94	0.707 NS
(NS- Not significant)
